# Combining ATRP and FRP Gels: Soft Gluing of Polymeric Materials for the Fabrication of Stackable Gels

**DOI:** 10.3390/polym9060186

**Published:** 2017-05-24

**Authors:** Antoine Beziau, Rafael N. L. de Menezes, Santidan Biswas, Awaneesh Singh, Julia Cuthbert, Anna C. Balazs, Tomasz Kowalewski, Krzysztof Matyjaszewski

**Affiliations:** 1 Department of Chemistry, Carnegie Mellon University, Pittsburgh, PA 15213, USA; antoine.beziau@gmail.com (A.B.); rafael.mnz1@gmail.com (R.N.L.d.M.); jcuthber@andrew.cmu.edu (J.C.); tomek@andrew.cmu.edu (T.K.); 2 Chemical Engineering Department, University of Pittsburgh, Pittsburgh, PA 15261, USA; santidanbiswas@gmail.com (S.B.); awaneesh11@gmail.com (A.S.); balazs@pitt.edu (A.C.B.)

**Keywords:** stacked gel, multi-layer gels, ATRP gel, FRP gel

## Abstract

Stackable gels comprised of layers of dissimilar polymers were synthesized by combining conventional free radical polymerization (FRP) and atom transfer radical polymerization (ATRP) using two approaches: (i) polymerization of a pre-gel solution containing a monomer and cross-linker introduced on top of a previously prepared gel, and (ii) simultaneous polymerization of two immiscible pre-gel solutions remaining in contact. All permutations of FRP and ATRP yielded single-piece, connected, amphiphilic gels regardless of the order of polymerization. Furthermore, multi-layer ATRP gels combining different polymers were synthesized with the FRP layer as a gluing agent. A 10-layer amphiphilic stackable gel combining *n*-butyl methacrylate (BMA) and 2-(dimethylamino)ethyl methacrylate (DMAEMA), and a 10-layer stackable gel combining BMA, DMAEMA and di(ethylene glycol) methyl ether methacrylate (PEO_2_MA) were synthesized. This patching method, combining conventional FRP gels with ATRP ones, offers an efficient path to the formation of complex stackable gel architectures.

## 1. Introduction

Polymer gels constitute an important class of soft materials due to their wide range of chemical and mechanical properties. Various gels have been synthesized using conventional free radical polymerization (FRP) of monovinyl and divinyl monomers. This technique has the following advantages: (i) a simple set up for large scale production and (ii) good tolerance to impurities. Moreover, the range of functional vinyl derivatives that can be used in FRP is broad, allowing for the selection of specific gel properties. For example, hydrogels made from methacrylic acid, *N*-*N*-dimethylacrylamide (DMA), 2-(dimethylamino)ethyl methacrylate (DMAEMA), *N*-isopropylacrylamide (NIPAM) or 2-hydroxyethyl methacrylate are used as superabsorbents [1,2], thermo-responsive materials [3], or biocompatible materials [4,5]. In addition, organogels made from *n*-butyl acrylate, *n*-butyl methacrylate, or other hydrophobic (meth)acrylate monomers are used as carriers of organic compounds for drug delivery [6,7].

Complex multi-layer hydrogels and organogels have also been prepared. For example, hybrid materials composed of two different gels were synthesized by polymerization of two high-viscosity pre-gel mixtures of DMA or NIPAM remaining in contact [8]. In another example, solutions of silica and cellulose nanoparticles were used to glue either dry or swollen poly(dimethylacrylamide) (PDMA) gels together, and dissimilar gels, such as PDMA and gelatin, together [9]. Yet another method to join dissimilar gels utilized rapid adhesion between poly(ethylene glycol) hydrogels swollen in water and organogels swollen in anisole, yielding macroscopic organo/hydrogel hybrids [10]. Acylhydrazone dynamic bonds formed by condensation of the aldehyde and acylhydrazine groups between the macroscopic gels acted as ‘gluing’ agents. This method allowed for reversible connections and relatively short reaction times, but it was applicable to a limited selection of monomers. Microgels were also connected using an electromicrofluidic platform [11].

The development of controlled radical polymerization (CRP) techniques such as atom transfer radical polymerization (ATRP) led to more precise control over polymer microstructures [12,13,14,15]. Gels produced by CRP were found to be more structurally homogeneous than their FRP counterparts [16,17,18,19,20,21,22,23,24,25,26,27,28]. Due to the fast initiation and intermittent deactivation of the propagating polymer chains, the fraction of terminated chains in such systems is low and the simultaneous growth of the chains prevents the formation of micro-gels in the early stage of the polymerization [18,29]. Furthermore, in ATRP, chain end functionality is preserved and the obtained polymers can be re-initiated [30,31]. This feature has been used to prepare block copolymers and multi-layered gels using the surface of one gel as a source of ATRP initiator for the polymerization of the next layer [32].

One of the limitations of using consecutive ATRP polymerizations in the preparation of multilayered gels, revealed in our past studies, was the poor connectivity between the two layers if the polymerization was carried out in immiscible solvents [33]. This limitation was not encountered when using the same solvent, dimethylformamide (DMF), for the two pre-gel solutions. This poor connectivity was attributed to the poor interfacial compatibility, preventing the formation of covalent cross-links and physical interactions between the two layers. It could be overcome using amphiphilic mikto-arm stars acting as interfacial linkers [33].

In this article, we demonstrate how the connectivity between dissimilar gels can be facilitated by alternating between ATRP and FRP in the preparation of consecutive layers. Specifically, layers prepared by the variant of ATRP with low ppm Cu catalyst, known as ICAR (initiators for continuous activator regeneration) ATRP were successfully glued together using interlayers prepared by FRP [34,35]. This combination of FRP and ATRP polymerization techniques offers an efficient strategy for the direct synthesis of flexible, multi-functional, and multi-layer materials. 

## 2. Materials and Methods 

### 2.1. Materials

2-(Dimethylamino)ethyl methacrylate (DMAEMA, 98%, Sigma-Aldrich, St. Louis, MO, USA), *n*-butyl methacrylate (BMA, 99%, Sigma-Aldrich, St. Louis, MO, USA), di(ethylene glycol) methyl ether methacrylate (PEO_2_MA), and poly(ethylene glycol) dimethacrylate (PEGDMA_750_, average molecular weight 750, Sigma-Aldrich, St. Louis, MO, USA), were passed through basic alumina column to remove radical inhibitors prior to use. Ethyl α-bromoisobutyrate (EBiB, 98% Aldrich), copper(II) bromide (99.999%, Aldrich), azo-initiators 2,2′-azobis[2-(2-imidazolin-2-yl)-propane] dihydrochloride (VA-044, Wako, Richmond, VA, USA), water (nanopure, 18.2 MΩ·cm^-1^, Thermo Fisher Scientific, Pittsburgh, PA, USA), 2,2′-azobis(4-methoxy-2,4-dimethylvaleronitrile) (V70, Wako, Richmond, VA, USA), rhodamine methacrylate (Polysciences Inc., Warminster, PA, USA), fluorescein methacrylate (Sigma-Aldrich, St. Louis, MO, USA), and *N*,*N*-dimethylformamide (DMF, ACS grade, Fisher Scientific, Pittsburgh, PA, USA) were used as received. Tris(pyridin-2-ylmethyl)amine (TPMA) and poly(ethylene glycol) α-bromoisobutyrate (PEG2KiBBr, average molecular weight 2000) were prepared as reported [36,37,38]. Glass slides (Borosilicate, McMaster-Carr, Atlanta, GA, USA) were treated with Rain-X^®^ anti-adhesive to prevent gel adhesion to the glass. Molds were made by placing a silicone rubber seal (extreme temperature silicone rubber^®^ 1 mm thick, McMaster-Carr, Altanta, GA, USA) between two glass plates.

### 2.2. Swelling Measurements

To characterize the swelling capability of each layer, a piece of the homo-polymer gel previously dried under vacuum and at room temperature for eight days, with weight ranging from 50 to 100 mg, was immersed in excess of solvent (water for PDMAEMA and toluene for PBMA) at room temperature (21 °C). After two days, the samples were weighed again. The swelling ratio was calculated as: $\mathrm{swelling}\;\mathrm{ratio}=\;\frac{w_{s}}{w_{d}}*100\%$, where *w_s_* and *w_d_* are the weights of the swollen and dry gels. This experiment was performed in triplicate. The range of the three values was reported as the uncertainty.

#### 2.2.1. Stackable Gel Synthesis: Solid/Liquid Sequential Procedure

A sequential stepwise solid/liquid method or a one pot liquid/liquid method was used to synthesize amphiphilic stackable gels. A patching method was also developed for the direct synthesis of multi-layer amphiphilic stackable gels and for the synthesis of multi-functional, multi-layer stackable gels. 

**Figure 1:**
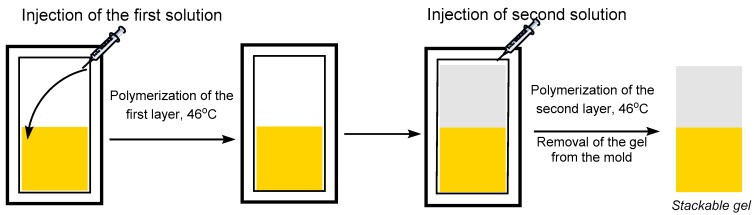
Solid/liquid sequential polymerization method.

The sequential polymerization procedure was performed as follows (Figure 1). A pre-gel solution was purged with nitrogen for at least 10 min and injected in a degassed mold using a syringe purged with nitrogen. Half the mold was filled under nitrogen flux and the mold was placed in an oven at 46 °C for 3 h to form the bottom gel layer. Then, the second pre-gel solution for the top layer was degassed for 10 min and injected into the mold under nitrogen. The fully filled mold was placed in the oven at 46 °C for 3 h. The mold was then opened and the gel was left in ambient air for 5 h before manipulation. The monomer conversion in the gels was determined in a separate polymerization experiment of each individual layer by NMR (Figure S1).

#### 2.2.2. Stackable Gel Synthesis: One Pot Liquid/Liquid Procedure

**Figure 2:**
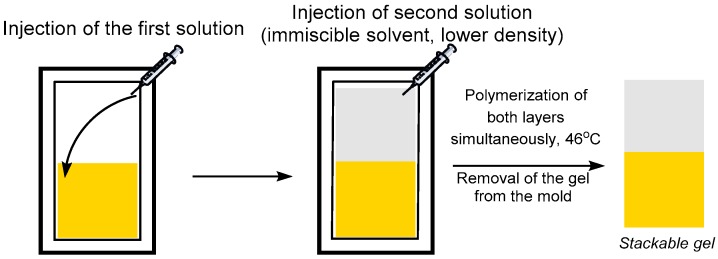
Liquid/liquid sequential polymerization method.

The one pot liquid/liquid method was performed as follows (Figure 2). The bottom and top layer pre-gel solutions were degassed separately for 10 min and injected sequentially into a previously degassed mold. The hydrophilic pre-gel solution based on DMAEMA in water was injected first and the hydrophobic pre-gel BMA solution in toluene was injected on top. Due to the solvent immiscibility, a biphasic pre-gel system was obtained and was polymerized in an oven at 46 °C (Figure S2).

### 2.3. Synthesis of n-Layer Stackable Gel Combining ATRP/FRP by Patching Method

**Figure 3:**
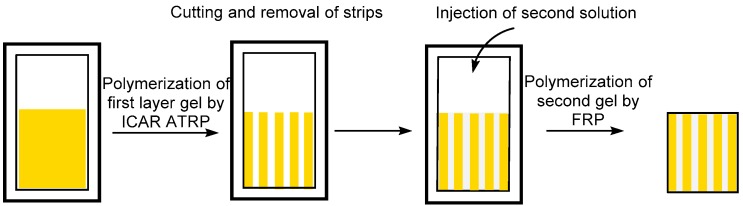
Patching method using FRP (free radical polymerization) glue to synthesize a 10-layer gel in one step.

The patching method consisted of attaching different layers (Figure 3). A polymer gel layer was synthesized in a mold by ATRP and pieces were cut and removed from the mold. The remaining pieces of gel were arranged in the mold, placed under nitrogen, the second FRP pre-gel solution was injected, and the mold was placed in an oven at 46 °C. For the synthesis of n-layer stackable gel combining different gel layers, the same method was used. For the synthesis of any other pattern, the ATRP gel was cut in the desired shape, placed into the mold, and the FRP pre-gel solution was injected (Figure S3). 

#### 2.3.1. Composition of the Pre-Gel Solutions

The layers were made by combining four different gels together made by FRP or ATRP: a DMAEMA based gel made by ICAR ATRP (DA), a BMA based gel made by ICAR ATRP (BA), a DMAEMA based gel made by FRP (DF), and a BMA based gel made by FRP (BF). For clarity, the order of polymerization will be reflected in the text. For example, DA/BF means that the stackable gel made from DMAEMA monomer by ICAR ATRP was polymerized first at the bottom and the gel made from BMA by FRP was polymerized second on top. The composition of the different pre-gel solutions is described in Table 1.

ATRP layers were made by ICAR ATRP. A solution of monomer, cross-linker, radical initiator, copper catalyst, and ATRP initiator was used as pre-gel solution and gelation was induced by decomposition of radical initiator in an oven set at 46 °C. The FRP layers were prepared similarly, but without ATRP initiator or copper catalyst/ligand. In order to visually differentiate the layers, an aqueous solution (50 μL of 10 mg·mL^−1^) of fluorescein methacrylate was copolymerized with the DA layers, and a DMF solution (50 μL of 10 mg·mL^−1^) of rhodamine methacrylate was copolymerized with the DF.

#### 2.3.2. Synthesis of PBMA Layer by FRP (BF)

The BF layer was prepared from a pre-gel solution containing BMA (1.06 g, 7.5 mmol), PEGDMA_750_ (375 mg, 0.5 mmol), and toluene (1.46 g). The solution was degassed under nitrogen for 10 min and then 1 mL was transferred to a vial containing the azo initiator V70 (8 mg, 0.026 mmol) previously degassed for 10 min. The reaction mixture was further degassed for 5 min. The solution was injected into a degassed mold and placed in an oven at 46 °C for 3 h. The initial molar ratio of reagents was as follows: [BMA]:[PEGDMA_750_]:[V-70] = 75:5:0.9; VM:VS = 1:1 in toluene. 

#### 2.3.3. Synthesis of PDMAEMA Layer by FRP (DF)

The DF layer synthesized by FRP was prepared from a prepolymer solution containing DMAEMA (1.18 g, 7.5 mmol), PEGDMA_750_ (375 mg, 0.5 mmol), and 1.75 g of distilled water. After 10 min of degassing under nitrogen, an aqueous solution (17 μL) of azo initiator VA-044 (60 mg/mL) was added to 1 mL of degassed solution. The reaction mixture was further degassed for 5 min. The solution was injected into the degassed mold and placed in an oven at 46 °C for 3 h. The initial ratio of reagents was as follows: [DMAEMA]:[PEGDMA_750_]:[VA-044] = 75:1:5:0.1:0.8:0.1; VM:VS = 1:1 in water. 

#### 2.3.4. Synthesis of PEO_2_MA Layer by ATRP (PA)

The PEO_2_MA stock solution was prepared from PEO_2_MA (Mn 300, 2.25 g, 7.5 mmol), PEG_2K_iBBr (200 mg, 0.1 mmol), pEGDMA_750_ (375 mg, 0.5 mmol), and 2.0 g of distilled water. To 1 mL of this stock solution, a DMF solution (150 μL) containing CuBr_2_ (20 mM) and TPMA (160 mM) was added. After 10 min of degassing under nitrogen, an aqueous solution (17 μL) of azo initiator VA-044 (60 mg/mL) and a DMF solution (50 μL) of rhodamine methacrylate (10 mg/mL) was added. The reaction mixture was further degassed for 5 min and was injected into the mold and placed in an oven at 46 °C for 3 h. The initial ratio of reagents was as follows: [PEO_2_MA]:[PEG_2K_iBBr]:[PEGDMA_750_]:[CuBr_2_]:[TPMA]:[VA-044] = 75:1:5:0.1:0.8:0.1; VM:VS = 1:1 in water.

## 3. Results and Discussion

### 3.1. Stackable Gels

The “stackable gel” concept is based on the sequential polymerization of two cross-linked polymer gels. It relies on forming (i) covalent inter-chain cross-links and (ii) physical interactions through surface contact and chain entanglement. Stackable gels combine the properties of two distinct gels into a single piece of material. For example, an amphiphilic stackable gel combines a hydrophilic water swollen layer and a hydrophobic oil swollen layer. 

The stackable gels prepared were synthesized using two different monomers: water soluble DMAEMA and hydrophobic BMA, which were polymerized in water and toluene, respectively. The different combinations of gel layers were synthesized by various permutations of FRP and ATRP polymerization techniques (Table 1). All gel polymerizations reached high conversion, >95% for the DA and DF layers, and >70% for the BA and BF layers. As determined by ^1^H NMR, gelation by FRP was faster than by ATRP (Figure S1). The DMAEMA polymerization by FRP in water reached the gel point after 4 min and, in the ATRP case, after 11 min. For the BMA polymerization in toluene, the BF layer gelled at 17 min, but the BA layer gelled after 2 h. The gelation time differences were not only due to solvents and radical initiators, but also due to the fact that in ATRP gelation occurs at the later stage of polymerization [19,29]. 

### 3.2. Formation of Amphiphilic Stackable Gels Combining ATRP and FRP by Solid/Liquid Sequential Polymerization

**Figure 4:**
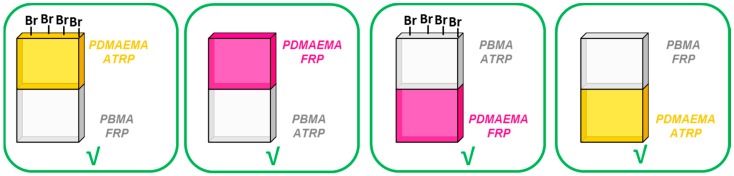
Stackable gels made by combining layers made by ATRP (atom transfer radical polymerization) and FRP using DMAEMA and BMA. The bottom layer was synthesized in a first step and the top layer is synthesized in a second step.

The synthesis of PDMAEMA/PBMA amphiphilic stackable gels by ATRP in a water/toluene system did not yield a well-connected stackable gel. However, when one of the layers was synthesized by FRP, a well-connected amphiphilic stackable gel formed. Four different combinations of FRP and ATRP stackable gels were investigated (Figure 4 and Figure S3). In every case, the polymerization of stackable FRP/ATRP hybrids was successful, independent of the order of polymerization. Since the reactant ratios and the solvent used were kept constant in both methods, the nature of the polymer networks facilitated the covalent interfacial connection. 

### 3.3. Formation of Amphiphilic Stackable Gels by Combining ATRP and FRP in a Liquid/Liquid One Pot Polymerization

In order to simplify the method and reduce the reaction time, a one pot method was developed. Since water and toluene are immiscible solvents and toluene (*d* = 0.87 g/cm^3^) is less dense than water (*d* = 1.0 g/cm^3^), bilayer pre-gel solutions—DF/BF, DF/BA, and DF/BF in water/toluene—were prepared and used in one pot polymerization (Figure S2). Simultaneous polymerization of both layers was also expected to improve the connectivity between the two gels by maximizing the surface contact at the interface.

Attempts to connect ATRP/ATRP amphiphilic layers with the water/toluene system failed, even when using a liquid/liquid method. This poor connectivity was in disagreement with the predictions of our previously reported simulations [39]. This discrepancy suggests that the phenomena responsible for connectivity occurred at a scale not captured by the simulations, which were limited to tens of nanometers. Some of those factors could include: entanglements, physical cross-linking, and difference in the kinetics of polymerizations (Figure S1) or the presence of short unattached chains (sol) in the ATRP system (Figure S4). Such unattached polymer chains were indeed detected by GPC after Soxhlet extraction from BMA gels prepared by ATRP, but were not present in the extracts from BMA gels prepared by FRP. It can be envisioned that migration of such unattached chains to the interface in ATRP/ATRP could prevent good connectivity between the layers.

### 3.4. FRP Gels as Soft Glues: Synthesis of Multi-Layer Gels by Patching Method

**Figure 5:**
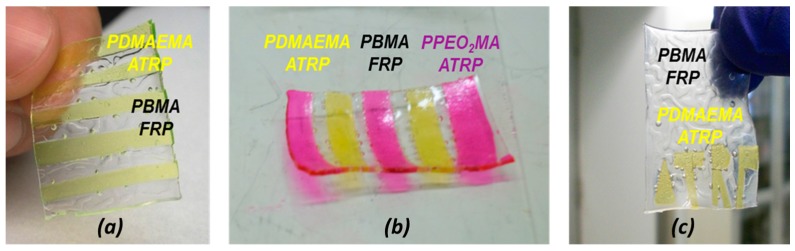
(**a**) Ten-layer gel prepared by connecting DMAEMA ATRP gel slices together using an FRP BMA gel. (**b**) Multi-layer gels obtained by connecting slices of a PEO_2_MA ATRP gel (containing RhMA) and DMAEMA ATRP gel (containing FluoMA) using BMA FRP gel as a soft glue. (**c**) “ATRP” embedded letter made of DMAEMA gel by ATRP and surrounding matrix made of BMA by FRP.

As described earlier, the BF layer efficiently connected to the ATRP layer regardless of whether it was polymerized as the first or second layer (Figure 5). Following this observation, the BF layer was then used as a ‘soft glue’ to synthesize multi-layered, amphiphilic stackable gels and multi-functional stackable gels incorporating three different type of polymeric gels. The ATRP layer(s) to be connected in this manner were synthesized either separately or in a mold. Three types of stackable gels were prepared by the patching method described previously (Figure 3): 10-layer stackable gel incorporating PDMAEMA layers by ATRP with PBMA layers by FRP (DA|BF|DA|BF|DA|BF|DA|BF|DA|BF gel) (Figure 5a).

- 10-layer stackable gel incorporating PDMAEMA and PEO_2_MA layers by ATRP and PBMA layers made by FRP (PA|BF|DA|BF|PA|BF|DA|BF|BF|PA|BF) (Figure 5b).
- “ATRP” letters formed by DA in a BF matrix (Figure 5c).

This procedure connected different hydrophilic ATRP layers together using the FRP hydrophobic layer as the glue, demonstrating that, not only different polymerization techniques, but also different gel properties can be combined in a single macroscopic gel. 

### 3.5. Influence of the Polymerization Technique: Swelling of the PBMA and PDMAEMA Gels

**Figure 6:**
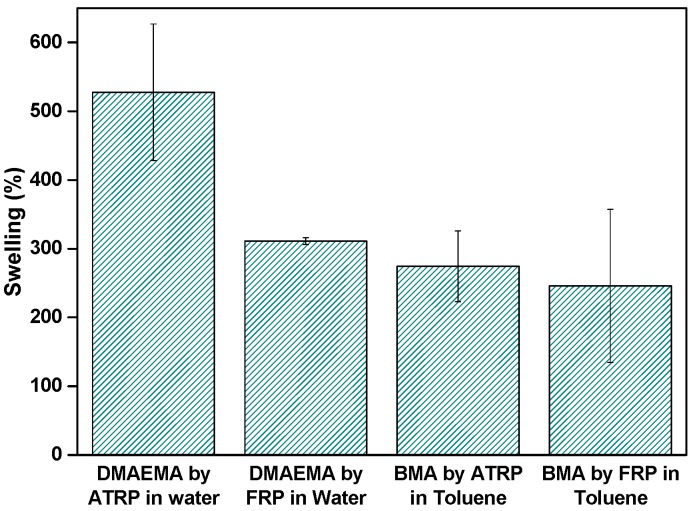
Swelling of the individual layers in water and toluene.

The polymerization technique significantly affected the swelling properties of the gel formed (Figure 6). In water, the DA gel swelling was 530% compared to the DF gel swelling of 310%. In toluene, the BA gel swelling was 270% compared to BF swelling of 240%. In all cases, the ATRP gels swelled more than FRP gels, indicating structural differences between the two types of networks. The ATRP network should have a more homogeneous structure, and, as such, is expected to accommodate more solvent molecules in comparison to the FRP network [40]. 

## 4. Conclusions

The stackable gels were successfully synthesized by combining ATRP and FRP layers. The amphiphilic, multi-layer gels were efficiently connected independent of the polymerization order. Previous simulations suggested that ATRP/ATRP stackable gels should connect covalently [39]. However, the GPC analysis of Soxhlet extract from ATRP gels showed the presence of unattached polymer chains (sol), which, after diffusing to the interface, could prevent covalent bonding between two layers prepared in immiscible solvents. To overcome this limitation, an FRP layer was used as a soft glue to connect multiple ATRP pieces together. Ten-layer, amphiphilic stackable gels, and 10-layer, tri-functional stackable gels were synthesized in a two-step procedure. The presented results further extend the stackable gel family and open the way for the fabrication of complex gel materials.

## Figures and Tables

**Table 1:** Composition of solutions used to prepare PDMAEMA or PBMA gels by ATRP or FRP.

Abb.	Gel	Method	Solvent	Rad. In.	[M]:[I]:[X]:[CuBr2]:[TPMA]:[Rad.In.] ^1^
DA	PDMAEMA	ICAR ATRP	Water	VA-44	75:1:5:0.1:0.8:0.1
BA	PBMA	ICAR ATRP	Toluene	V-70	75:1:5:0.1:0.8:0.9
DF	PDMAEMA	FRP	Water	VA-44	75:0:5:0:0:0.1
BF	PBMA	FRP	Toluene	V-70	75:0:5:0:0:0.9

^1^ “M” refers to monomer, “I” refers to ATRP initiators, “X” refers to the cross-linker, and “Rad. In.” refers to the radical initiator. For all solutions, V_solvent_/V_total_ = 0.5. In the ATRP polymerization, an ATRP initiator, CuBr2, and TPMA ligand are additional reactants.

## Supplementary Materials

The following are available online at www.mdpi.com/2073-4360/9/6/186/s1, Figure S1: The kinetic study was performed as follows: 3 mL of the pre-gel solution degassed with nitrogen for 20 min and was injected to a Schlenk flask previously degassed for 20 min containing the radical initiator. The system was maintained at 45 °C for 3 h and conversion was followed by NMR. After gelation, only the last point (at 3 h) was taken. A piece of the gel formed (ca. 350 mg) was extracted with deuterated DMSO for 6 h and analyzed by NMR using the DMF peak as the internal standard. (A) PDMAEMA gel made by FRP (DF); (B) PDMAEMA gel made by ATRP (DA); (C) PBMA made by FRP (BF); (D) PBMA made by ATRP (BA). Figure S2: The liquid/liquid procedure where both pre-polymer solutions were injected sequentially into the mold. Figure S3: Example pictures of different stackable gels. Figure S4: GPC trace of the polymer after Soxhlet extraction from BMA gels.
