# Supplementary material for: Combining ATRP and FRP Gels: Soft Gluing of Polymeric Materials for the Fabrication of Stackable Gels
Source: Polymers (Basel). 2017 May 24;9(6):186. doi: 10.3390/polym9060186 (PMC6432409; doi:10.3390/polym9060186)
Supplement: Supplementary file 1 [file polymers-09-00186-s001.pdf]

Supplementary Materials: Combining ATRP and FRP Gels: Soft Gluing of Polymeric Materials for the Fabrication of Stackable Gels

Antoine Beziau, Rafael N. L de Menezes, Santidan Biswas, Awaneesh Singh, Julia Cuthbert, Anna C. Balazs, Tomasz Kowalewski, and Krzysztof Matyjaszewski

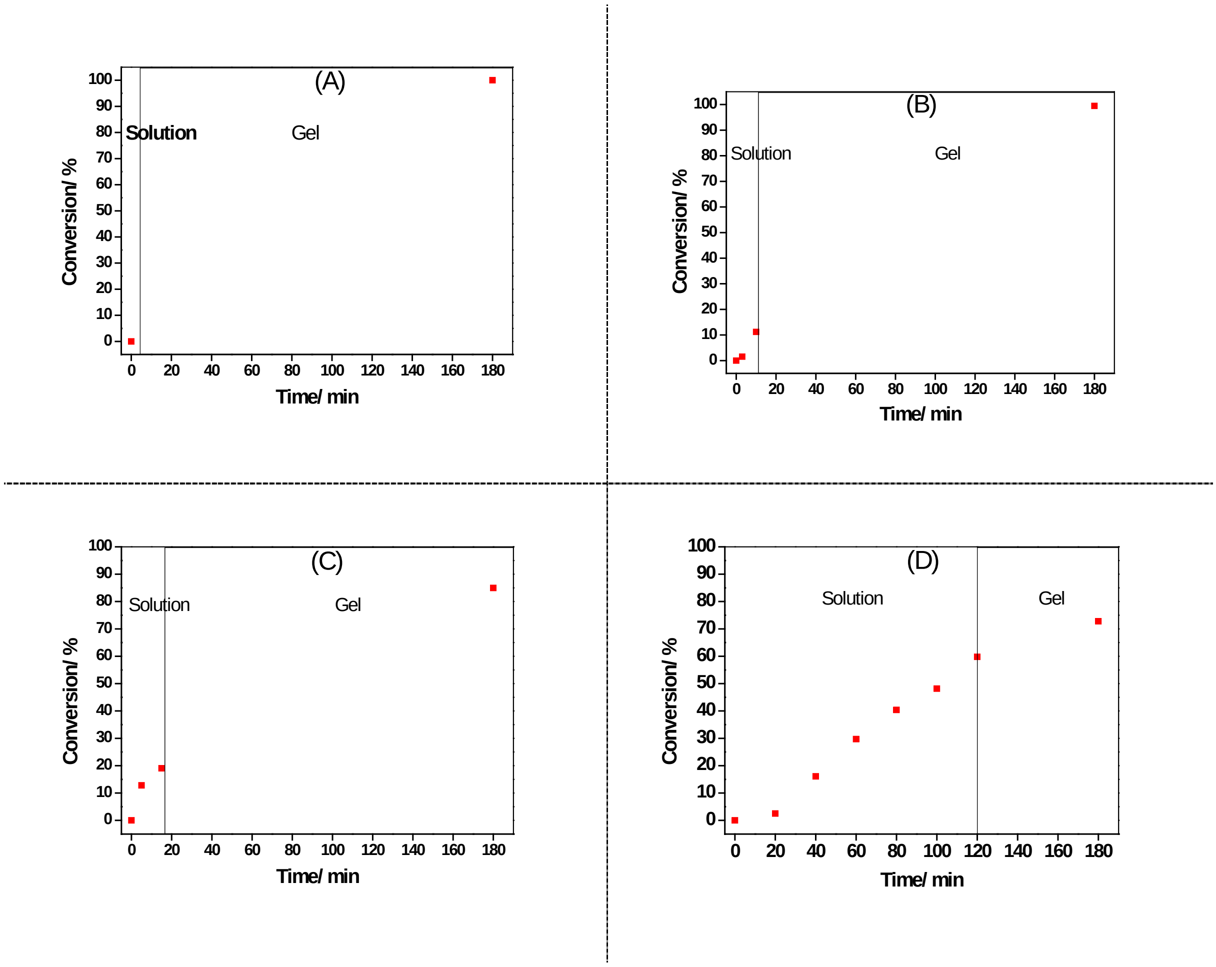

Figure S1. The kinetic study was performed as follows: 3 mL of the pre-gel solution degassed with nitrogen for 20 minutes and was injected to a Schlenk flask previously degassed for 20 minutes containing the radical initiator. The system was maintained at 45 °C for 3 h and conversion was followed by NMR. After gelation, only the last point (at 3 h) was taken. A piece of the gel formed (ca. 350 mg) was extracted with deuterated DMSO for 6 h and analyzed by NMR using the DMF peak as the internal standard. A) PDMAEMA gel made by FRP (DF); B) PDMAEMA gel made by ATRP (DA); C) PBMA made by FRP (BF); D) PBMA made by ATRP (BA).

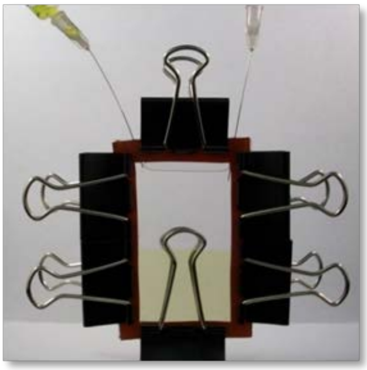

Figure S2. The liquid/liquid procedure where both pre-polymer solutions were injected sequentially into the mold. The bottom layer in yellow is the DMAEMA layer in water, the transparent layer on the top is the BMA layer in toluene.

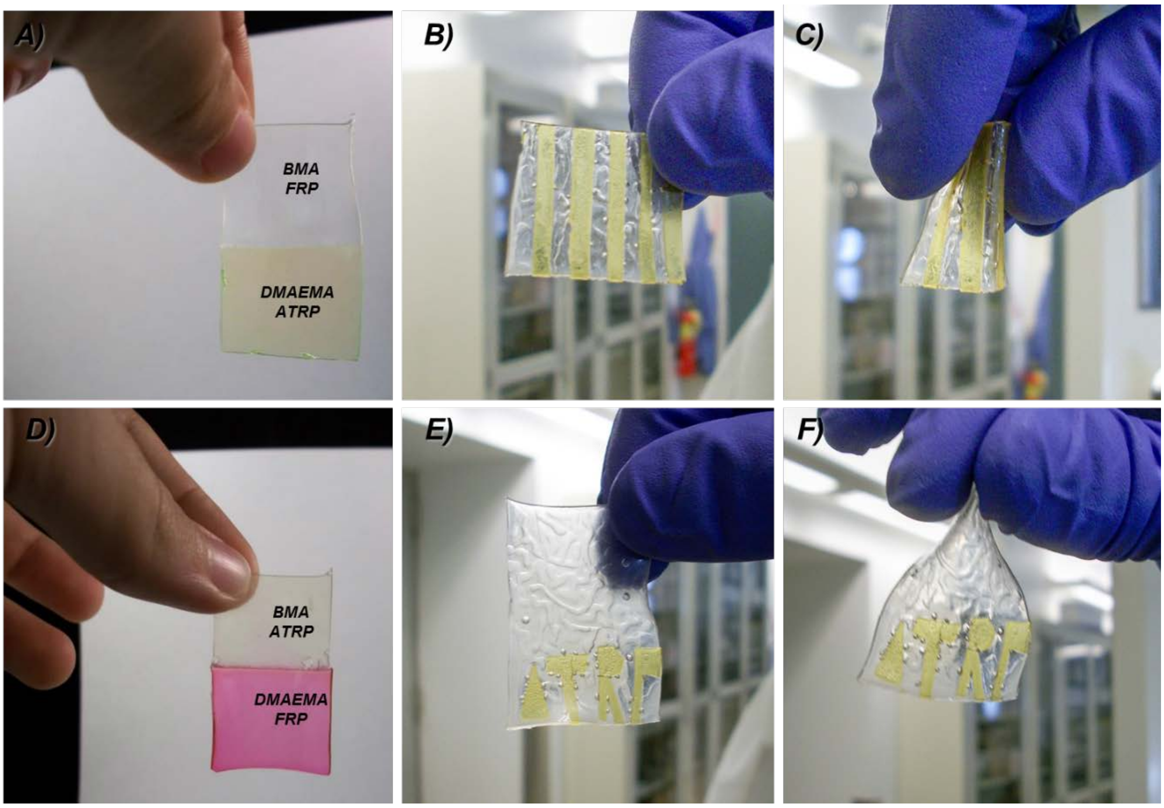

Figure S3. Example pictures of different stackable gels. A) DA/BF layer stackable gel; B) and C): Images of a 10-layer stackable gel D) BF/DA stackable gel; E) and F) Patterned gel.

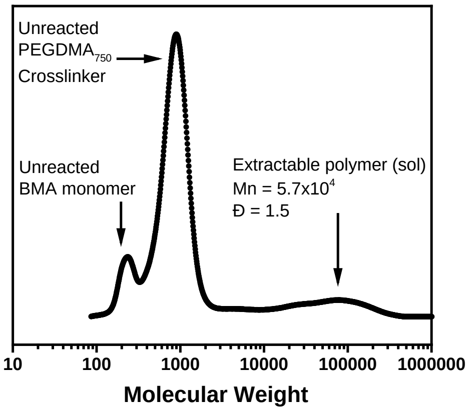

Figure S4. GPC trace of the polymer after Soxhlet extraction from BMA gels. Gel samples (70 mg) made by ATRP were extracted in dichloromethane and analyzed by GPC, showing unattached chains not incorporated into the gel network (sol). For the FRP gels, with the same procedure, no polymer was detected.
